# Seeking Support on Facebook: A Content Analysis of Breast Cancer Groups

**DOI:** 10.2196/jmir.1560

**Published:** 2011-02-04

**Authors:** Jacqueline L Bender, Maria-Carolina Jimenez-Marroquin, Alejandro R Jadad

**Affiliations:** ^3^Department of Health Policy, Management and EvaluationUniversity of TorontoToronto, ONCanada; ^2^Dalla Lana School of Public HealthUniversity of TorontoToronto, ONCanada; ^1^Centre for Global eHealth InnovationUniversity Health NetworkToronto, ONCanada

**Keywords:** Internet, Facebook, breast cancer, supportive care, peer support, health promotion, fundraising

## Abstract

**Background:**

Social network sites have been growing in popularity across broad segments of Internet users, and are a convenient means to exchange information and support. Research on their use for health-related purposes is limited.

**Objective:**

This study aimed to characterize the purpose, use, and creators of Facebook groups related to breast cancer.

**Methods:**

We searched Facebook (www.Facebook.com) using the term breast cancer. We restricted our analysis to groups that were related to breast cancer, operated in English, and were publicly available. Two of us independently extracted information on the administrator and purpose of the group, as well as the number of user-generated contributions. We developed a coding scheme to guide content analysis.

**Results:**

We found 620 breast cancer groups on Facebook containing a total of 1,090,397 members. The groups were created for fundraising (277/620, 44.7%), awareness (236, 38.1%), product or service promotion related to fundraising or awareness (61, 9%), or patient/caregiver support (46, 7%). The awareness groups as a whole contained by far the most members (n = 957,289). The majority of groups (532, 85.8%) had 25 wall posts or fewer. The support oriented groups, 47% (27/57) of which were established by high school or college students, were associated with the greatest number of user-generated contributions.

**Conclusions:**

Facebook groups have become a popular tool for awareness-raising, fundraising, and support-seeking related to breast cancer attracting over one million users. Given their popularity and reach, further research is warranted to explore the implications of social network sites as a health resource across various health conditions, cultures, ages, and socioeconomic groups.

## Introduction

Online communities present a convenient means to exchange information and support with people in similar circumstances and are increasingly being used for health purposes [1], particularly by breast cancer survivors [2]. One of the most popular and perhaps most successful online communities, if success is based on sheer numbers of registered users, is the social network site Facebook (www.Facebook.com). Just over 5 years since its launch, Facebook became the second most visited website in the world (second only to Google) [3], with over 500 million active users (those who returned to the site within the last 30 days) worldwide [4]. While young adults are still more likely to use social network sites [5], the fastest growing demographic of Facebook users is women 55 years and older [6], which corresponds to the average age of onset of breast cancer [7]. Although recent studies indicate that Facebook groups are used for health purposes [8], little is known about how this resource is used by people affected by breast cancer.

Online communities are “virtual social space(s) where people come together to get and give information or support, to learn or to find company” [9]. They tend to be characterized according to the activity (eg, support) or the people that they serve (eg, breast cancer survivors), or the communication technology that supports them (eg, message board) [10]. Initially, online communities were supported by mailing lists, and asynchronous and synchronous message boards. More recently online communities have formed around blogs, wikis, and social network sites, commonly referred to as Web 2.0 social media applications [11]. Social network sites are differentiated from other online communities based on their ability to enable users to display their social networks. Their backbone consists of visible user profiles that display an articulated list of friends who are also users of the system [12]. While other online community platforms enabled users to create a list of friends, these networks were not displayed or accessible to other users. This unique feature of social network sites is hypothesized to result in connections between individuals that would not otherwise have been made [12].

Research on online communities for health purposes has primarily focused on the use and effects of mailing lists and message boards by breast cancer survivors, who have been shown to be one of the groups most likely to seek support from peers on the Internet [2]. Qualitative studies have revealed that these types of online communities provide breast cancer survivors with a safe, relatively anonymous space to communicate about sensitive and potentially stigmatizing topics [13], reduce feelings of isolation and uncertainty regarding prognosis and ambiguous painful symptoms [14], and enable them to become more informed and better prepared for their interactions with the health system [15]. Randomized controlled trials have shown that professionally moderated mailing lists and message boards for breast cancer survivors can reduce depression, stress, and cancer related trauma, and can enhance social support [16-18].

Relatively little is known about the use of social network sites for health purposes. Keelan and colleagues [19,20] examined the use of YouTube videos and Myspace blogs as a source of information on immunization and found a subcommunity of users critical of or with divergent views about vaccines. Research by Scanfeld and colleagues has demonstrated that Twitter has been used to share information on the use and side effects of antibiotics [21]. To our knowledge, there is only one study of the use of Facebook for health purposes. Farmer et al [8] examined noncommunicable disease groups and found a considerable number of patient and caregiver support groups related to malignant neoplasms. Surprisingly, breast cancer groups were notably absent from their analysis.

Breast cancer is the most common cancer among women worldwide [22], and thanks to advances in detection and treatment, women affected by this disease form the largest group of female cancer survivors [23]. However, the posttreatment period carries numerous physical and psychosocial needs that often go unaddressed by professional health care services [23]. Addressing the needs of this growing population of cancer survivors has been identified as supportive care’s new challenge [23,24]. Social network sites could provide breast cancer survivors with a convenient means to connect with a diverse network of peers, thus facilitating access to a wider array of supportive information and services. In fact, some have questioned the utility of government-funded personal health care solutions, when social network sites provide users with the tools to create and share health resources on their own [25]. Little is known about how people affected by breast cancer use social network sites. This study attempted to fill some of the gaps by presenting a characterization of the purpose, patterns of use, and creators of Facebook groups related to breast cancer.

## Methods

### Search Strategy

**Figure 1:**
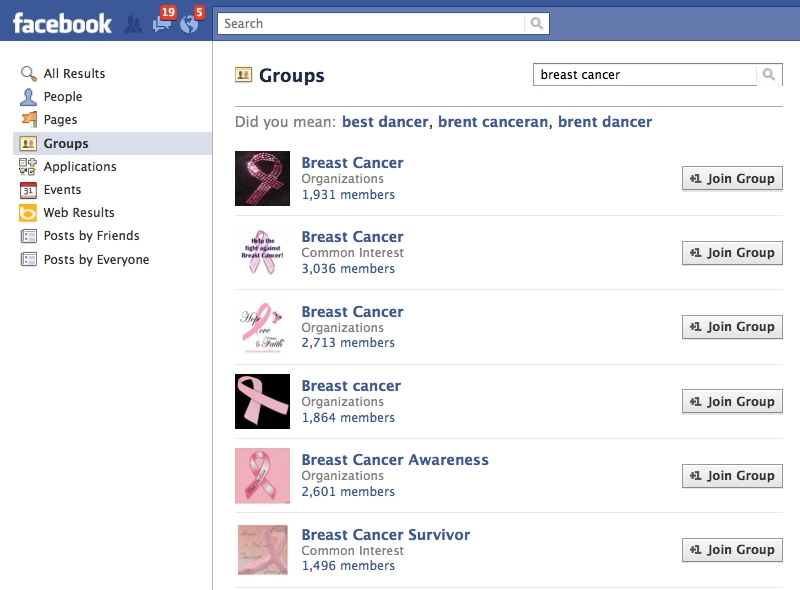
Sample Facebook search result restricted to group pages

On November 19, 2008 we searched Facebook using the platform’s built-in search engine and the keyword breast cancer (Figure 1). We restricted our analysis to Facebook groups that were related to breast cancer, operated in English, and were publicly available to anyone with a Facebook account to view and join. Pages for individual members, organizations, events, and applications were excluded.

### Data Extraction

Two of us (JLB and MCJM) independently reviewed the resulting set of eligible groups and extracted information on the following: (1) general characteristics (eg, group name, purpose, creator, and URL), and (2) membership and user-generated content (eg, number of members, discussion posts, wall posts, photos, and videos).

### Data Analysis

We determined the purpose of each group based on a content analysis of, and in order of priority (if available), the title of the group, the description of the group, the information in the Recent News section, the discussion posts, and the wall posts. (The content analysis of the discussion and wall posts was restricted to those displayed on the main page of the group.)

We began by analyzing the content of the first 100 groups to develop a coding and classification scheme that could be applied to the entire set. This initial step led to the identification of four main types of breast cancer groups:

- Fundraising groups: created to attract financial resources for breast cancer through an event, product, or service. Visitors to these groups were asked to donate money, or to purchase a product or ticket to an event. Instructions were typically provided regarding how or where to donate the funds.
- Awareness-raising groups: created to bring attention to the importance of breast cancer in general, or to promote a charitable organization, a fundraising event, or screening or research program.
- Support groups: created to meet the informational and emotional needs of breast cancer survivors or affected family members or friends.
- “Promote-a-site” groups: created to increase the prominence of an external website raising funds or awareness for breast cancer through the sale of products or services.

After independently classifying the general purpose of the groups using the above coding scheme, we resolved any differences. Next we generated a second-tier coding scheme to subclassify and more specifically describe the purpose of each group.

We also developed and independently applied a coding scheme to classify the approximate age and geographic location of the creators of the support groups. We restricted our analysis of the creators to the support groups, because we were primarily interested in the role of Facebook groups as a source of supportive care.

Lastly, we calculated descriptive statistics using SPSS version 17 (IBM Corporation, Somers, NY, USA) to summarize and compare the size (in terms of number of members) and amount of user-generated contributions of each type of group (in terms of wall posts). Most data were expressed as medians with interquartile ranges (IQRs) because the number of group members and user-generated content varied considerably and did not follow a normal distribution. We used chi-square tests to compare categorical data across groups.

This study was a component of a larger research study for which ethical approval was obtained. However, it should be noted that this study met the exclusion criteria of the (Canadian) Tri-Council Policy Statement as to what studies require review by an institutional research ethics board, because all information was publicly available.

## Results

**Figure 2:**
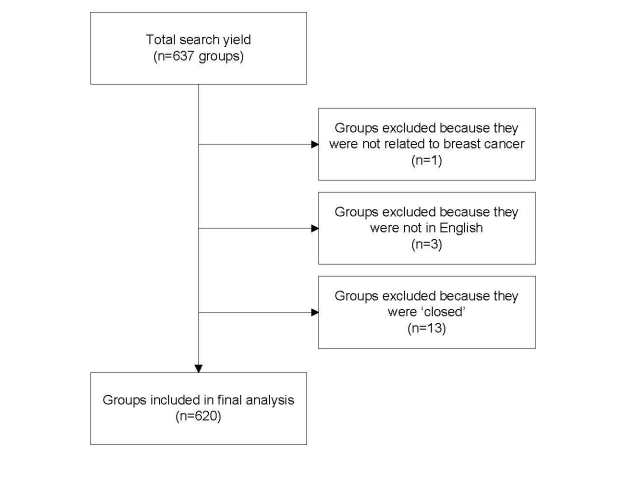
Flow diagram of group selection process

**Figure 3:**
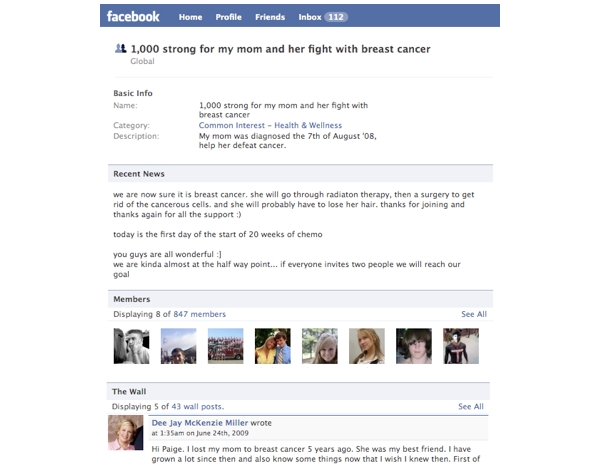
Sample breast cancer support group on Facebook in 2008

**Figure 4:**
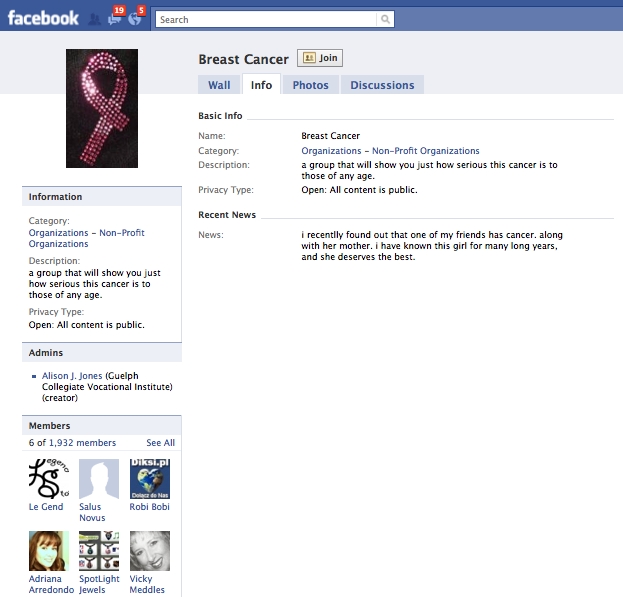
Sample breast cancer awareness group on Facebook in 2010

The search of Facebook on November 19, 2008 yielded 637 groups. As shown in Figure 2 620 groups were included in the final analysis. We excluded one group because it was not related to breast cancer, three groups because they were not in English, and 13 groups because they were “closed.” Figure 3 shows an example of a breast cancer support group on Facebook at the time the study was conducted. Since then, the platform has undergone revision, including changes to the way information is displayed on the group pages and the addition of new features (eg, group chat). Figure 4 shows an example of the current layout of a breast cancer awareness group on Facebook.

### Group Purpose

 As shown in Table 1, the majority of groups (513/620, 82.7%) were created for fundraising or awareness purposes. In total, 44.7% (277/620) were created to raise funds for breast cancer, 38.1% (236/620) to raise awareness about breast cancer and related events, 10% (61/620) to promote an external website raising funds or awareness for breast cancer through the sale of products or services, and 7% (46/620) to generate support for people affected by breast cancer. A minority of groups (9%) were classified as having an additional purpose, 34% (19/55) of which related to fundraising or support, 27% (15/55) to raising awareness, and 4% (2/55) to supporting an external website. As shown in Table 2, the three most common types of breast cancer groups on Facebook, which comprised 69% of the total sample, were (1) groups created to raise funds for a fundraising walk associated with a charitable organization in the United States or Canada (239/620, 38.5%), (2) groups raising awareness about a specific fundraising event (95/620, 15%), or (3) groups promoting the importance of breast cancer in general (94/620, 15%).

**Table 1:** General purpose and size of Facebook breast cancer groups

Group	Sample group description	n (%)	Members			
			Total	Median (IQR)^a^	Minimum	Maximum
Fundraising	*My mom is a 11 yr cancer survivor and i [sic] am walking for her and encouraging friends and family to join me in this walk for a cure for breast cancer.*	277 (44.7)	51,307	151 (92)	1	2623
Awareness	*October is Breast Cancer Awareness Month. Share Beauty...Spread Hope ...Think Pink!!*	236 (38.1)	957,289	270 (389)	2	772,815
Promote-a-site	*This doesn’t cost you a thing. Their corporate sponsors/advertisers use the number of daily visits to donate a mammogram in exchange for advertising.*	61 (10)	64,861	373.5 (932)	116	16,769
Support	*For anyone who knows someone who has survived, is battling, or has died of breast cancer. For congratulations, hope and [in] memoriam.*	46 (7)	16,940	235.5 (237)	2	2995

^a^ IQR: interquartile range.

**Table 2:** Specific purpose and frequency of Facebook breast cancer groups

General purpose	Specific purpose	Primary purpose, n	Secondary purpose, n
Fundraising (n = 277)	1. Charity fundraising event	239	2
	2. Personal fundraising event	11	14
	3. Product promotion	17	0
	4. Charitable organization	7	2
	5. Noncharitable organization event	3	0
	6. Service promotion	0	1
Awareness (n = 236)	1. Breast cancer in general	94	7
	2. Fundraising event (eg, walk)	95	6
	3. Charitable organization	23	1
	4. Awareness event	10	1
	5. Research project	5	0
	6. Political advocacy	4	0
	7. Risk factors	3	0
	8. Planning an event	2	0
Promote-a-site (n = 61)	1. Product promotion	43	2
	2. Political advocacy	16	0
	3. Awareness	1	0
	4. Research recruitment	1	0
Support (n = 46)	1. For anyone affected by breast cancer	22	10
	2. For oneself or loved one with breast cancer	22	3
	3. For fundraisers	2	6
Total		620	55

### Group Size

We identified a total of 1,090,397 Facebook users who were members of one or more of the 620 breast cancer groups. The awareness groups contained by far the most members (957,289, 87.8%), followed by the promote-a-site groups (64,861, 5.9%), fundraising groups (51,307, 4.7%), and support groups (16,940, 1.5%). The groups ranged in size from 1 to 772,815 members and had a median of 196.5 members (IQR 214.7). Most groups (612/620, 98.7%) contained 5000 or fewer members and 70.8% (439/620) contained 101 to 500 members. On average, the promote-a-site groups had the greatest median number of members (median 373.5, IQR 932), followed by the awareness groups (median 270, IQR 389), support groups (median 235.5, IQR 237), and fundraising groups (median 151, IQR 92) (Table 1).

### User-Generated Contributions

A user can contribute content to a Facebook group in various ways, such as posting messages to the “wall,” news section, or discussion board, or uploading multimedia such as photos or videos. As Table 3 shows, the most frequently used communication feature was the wall. Although wall posts ranged in number from 0 to 8614, the groups contained a median of 5 wall posts (IQR 11). The majority of groups (532/620, 85.8%) had 25 wall posts or fewer. The support groups had the greatest median number of wall posts (median 16, IQR 38), followed by the awareness groups (median 6, IQR 19), promote-a-site groups (median 4, IQR 9), and fundraising groups (median 4, IQR 7). The difference in median number of wall posts across the groups was statistically significant (c^2^_3_= 52.0, *P* < .001). 

**Table 3:** User-generated content on Facebook breast cancer groups, median (interquartile range)

Group	Wall posts	Discussion posts	Photos	Videos
Support	16 (38)	1 (4)	3 (12)	0 (0)
Awareness	6 (19)	1 (3)	3 (11)	0 (0)
Fundraising	4 (7)	0 (1)	0 (6)	0 (0)
Promote-a-site	4 (9)	2 (2)	0 (1)	0 (0)

### Support Groups

Nearly half (32/65, 49%) of the support groups were created to generate support for anyone affected by breast cancer. A typical purpose statement for these types of groups was “For anyone who knows someone who has survived, is battling or has died of breast cancer. For congratulations, hope and [in] memoriam.” An additional 38% (25/65) of the support groups were established to obtain support for the creator of the group or a loved one affected by breast cancer and 12% (8/65) were created as a forum for information sharing among people participating in a fundraising walk (Table 2). Interestingly, a minority of the groups that were created “for anyone” affected by breast cancer (6/32, 19%) were initiated by individuals with an afflicted family member or friend, even though the explicit purpose of the group was not to gain support for the creator of the group or a loved one in particular. In the remaining 26 of these groups, the motivation of the group creator was not explicitly described. A small percentage of the support groups (5/65, 8%) were also serving in memoriam of a loved one who had died of breast cancer.

### Support Group Creators

We also examined the creators of the support groups for anyone, oneself, or a loved one affected by breast cancer (excluding groups created as a support forum for people participating in a fundraising walk, because we were primarily interested in breast cancer-related support). All but one of the creators of the support groups (n = 57) restricted the visibility of their personal profile pages to members within their networks. However, in 47% (27/57) of the support groups the academic institution of the creator and their expected graduation date either was included on the group page itself or was available in the search result content, and in 86% (49/57) of the support groups the geographic location of the creator was also available. Of the groups with available information on the approximate age of the group creators, 56% (15/27) were college students, 37% (10/27) were high school students, and 7% (2/27) were recent college graduates. None of the support group creators appeared to be health care professionals or associated with a health care organization. Of the groups with available information on the geographic location of the support group creators, 57% (28/49) were located in the United States, 41% (20/49) in Canada, and 2% (1/49) in Australia.

## Discussion

We found a large number of breast cancer-related groups on Facebook (n = 620) with over one million members. Unlike most disease-specific online communities, the majority of breast cancer groups on Facebook were created for fundraising and awareness purposes, rather than supportive care. The awareness groups as a whole contained by far the most members (n = 957,289), while the support groups were associated with the greatest number of user-generated contributions. Many of the individuals who did create the groups for supportive care purposes were adolescents and young adults, and the majority appeared to be living in the United States or Canada. None of the support group creators appeared to be health care professionals or associated with a health care organization.

Unlike in our study, Farmer et al [8] found patient (47.4%) and caregiver support groups (28.1%) to be more common than fundraising groups (18.6%). However, Farmer et al did not include breast cancer groups in their sample. Of relevance, the authors did include lung, stomach, and colorectal cancer as search terms, and found considerably fewer groups (n = 55) and members (n = 77,832) associated with these neoplasms, than we found associated with breast cancer (620 groups with 1,090,397 members). This difference is largely due to the greater number of fundraising and awareness groups we found associated with breast cancer, which is not surprising given that the breast cancer fundraising movement is one of the largest and most successful survivor-driven social movements, which other disease groups seek to emulate [26]. However, we also found more support groups for breast cancer (n = 47) than Farmer et al found for lung, stomach, and colorectal cancer combined (n = 32). Although breast cancer is the most common neoplasm in women, lung, stomach, and colorectal cancers are the three neoplasms associated with the greatest morbidity and mortality among both men and women worldwide [22]. Hence, the difference in the number of support groups on Facebook associated with these cancers cannot be attributed to their relative prevalence, and may instead reflect a greater tendency for people affected by breast cancer to join online communities than people affected by other conditions [2].

In contrast to breast cancer-specific online communities, which are used primarily to meet treatment information, symptom management, and emotional support needs [27], breast cancer groups on Facebook were not primarily used for supportive care purposes. One of the frequently reported advantages of breast cancer-specific online communities, which to date have focused on mailing lists and message boards, is the relative anonymity and privacy that they provide, which allows users to communicate about personal and socially stigmatizing topics [13]. Although Facebook groups provide facilities for discussion forums based on shared experiences, the visibility of user profiles and personal networks reduces the relative anonymity of the encounter and, if open to the public, which all groups in this study were, they have the potential to attract a much wider audience. This core functionality of social network sites, which gives users access to a more diverse and extensive network, makes them ideally suited for fundraising and awareness-raising purposes, as this study has demonstrated, but may make them less suitable for support-seeking related to topics that are embarrassing or socially stigmatizing [2].

Many of the individuals who did create the groups for supportive care purposes were adolescents and young adults, and the majority appeared to be living in the United States or Canada. These findings reflect the site’s user demographics at the time study was conducted. In the fall of 2008, the largest demographic of Facebook users was 18-24 years old [5], the United States reported more Facebook users than any other country, and Canada had the highest penetration of Facebook users per capita [28]. While some support groups were created for a loved one affected by breast cancer (perhaps a less technology-savvy parent), many young people established Facebook groups to obtain support for themselves.

Adolescents and young adults can experience significant distress when a loved one has cancer [29,30], and research suggests that their unique needs are often poorly met both within and outside the family [31]. Social network sites such as Facebook could provide this group with a convenient and familiar means to accumulate coping resources. Use of these sites is associated with greater levels of *bridging social capital,* or access to information and resources through a diverse set of acquaintances, and *bonding social capital,* or emotional support from close friends [32]. Both of these, according to the theory of stress and coping, can promote coping efforts and lessen negative appraisals of events, in turn reducing or buffering anxiety [33]. Furthermore, Ellison et al [34] have shown that college students who are active on Facebook experience higher levels of both forms of social capital, and Burke and colleagues [35] have confirmed that these findings generalize to older users and English speakers outside the United States.

Notwithstanding the large number of members that the breast cancer groups attracted, there were relatively few user contributions overall, and in the fundraising, awareness, and promote-a-site groups in particular. These findings support the consistently reported observation that online communities attract significantly more lurkers (visitors who do not post messages) than posters [36]. However, the fundraising, awareness, and promote-a-site groups were not created to stimulate discussion but rather to promote a message, event, product, or service. Although activity, which is often judged by the number of posts, is a key determinant of a successful online community [37], posting messages in online health communities is not necessary to obtain the empowering effects from participating in them [38]. Likewise, it may be possible to benefit from joining a Facebook group without contributing content, depending on the purpose of the group or the motivation of the joiner. According to a study by Park et al [39], college students join Facebook groups not just to socialize, but also to obtain information about events, to seek self-status, and to find entertainment. In addition, Park and colleagues found that those who joined Facebook groups for information purposes were more likely to participate in civic and political activities, suggesting that Facebook groups may play an important role in facilitating youth engagement.

### Practice Implications

The findings of this study are valuable because they provide information on the health-related use of the most widely popular social network site in existence. They indicate that Facebook groups are being used by a considerable number of people affected by breast cancer for fundraising and awareness purposes, and to a lesser extent supportive care. That being said, our findings should not be interpreted to imply that Facebook is rarely used for supportive care purposes, given that several ways to solicit or provide support on Facebook were not examined in this study, including private messages, wall posts on personal profile pages, and status updates. These findings do suggest that Facebook may play an important role in facilitating public engagement in health promotion and fundraising activities, particularly among youth.

### Limitations

This study has important limitations. First, we were unable to collect demographic information on 53% (30/57) of the support group creators due to their use of privacy settings. However, this finding suggests that users of Facebook not only are becoming aware of the public nature of their online activities, but also are activating the privacy measures offered. In fact, all but one of the support group creators in our sample restricted their personal Facebook profiles to their networks, whereas a study of Facebook users conducted in 2005 found that only 0.06% of college students restricted the visibility of their profiles to members within their networks [40]. Since then, significant changes made to the platform and user base of Facebook might in part explain the increased use of privacy settings by this sample, such as the launch of the NewsFeed feature, which provides updates on the activities of friends [41], the introduction of third-party-developed applications [42], and the expansion of registration to anyone.

Another related limitation was our reliance on user self-reported data (that were available on the group page itself or in the search result content) to infer the approximate age and geographic location of the support group creators. This information is possibly incorrect or fabricated. In addition, we could not determine the exact number of unique individuals affiliated with a particular type of breast cancer group on Facebook, given that a single user could be a member of multiple groups. Therefore, the total number of members affiliated with each type of breast cancer group could be inflated. At the same time, the total number of breast cancer groups identified in this study is likely only a portion of the total number of breast cancer groups on Facebook, given that we restricted our study to groups in English, while Facebook is available in more than 70 different language versions [4].

Lastly, we encountered numerous challenges while investigating the nature of breast cancer groups on Facebook that were primarily related to its limited functionality as a search tool. The search bar yields an imprecise yield (eg, “>500 groups”), the order of the search results is inconsistent and unclear, and the search is limited to the title of the group. Since the time we conducted our study the search tool has been enhanced but, to our knowledge, these specific issues have yet to be resolved. We contacted Facebook to notify them of these technical issues and obtained an encouraging response. Collaboration with platform owners would certainly facilitate future research in this area. 

### Research Implications

Further research is warranted to understand the implications of participating in health-related groups on Facebook. While other researchers have examined site activities that lead to higher levels of social capital [34,35], no known studies have examined the impact of participating in a health-related group on Facebook. It is also unknown whether general social network sites such as Facebook are as effective as disease-specific online communities in providing health-related information and support, and for whom. Given the importance of anonymity in facilitating disclosure in online breast cancer communities [13], research is warranted to examine breast cancer survivors’ perceptions of social network sites as a source of supportive care in comparison to other sources. Lastly, a better understanding is needed of the privacy implications of sharing personal health information on public social network sites, which has raised concern [25], leading some to advise against disclosing personal information on these sites [8].

### Conclusions

Facebook groups have become a popular tool for awareness-raising, fundraising, and support-seeking related to breast cancer, attracting over one million users by the end of 2008. Given their popularity and reach, further research is warranted to explore the implications of social network sites as a health resource across various health conditions, cultures, ages, and socioeconomic groups.

## Abbreviations

IQR: interquartile range
